# Environment sensing technology of underwater ROV based on artificial siding

**DOI:** 10.1371/journal.pone.0304226

**Published:** 2025-07-23

**Authors:** Wenhui Wang, Fumeng Ye, Rufei He

**Affiliations:** CSG PGC Power Storage Research Institute, Guangzhou, China; Chitkara University Institute of Engineering and Technology, INDIA

## Abstract

The underwater environment has the characteristics of high pressure and low temperature, which limits the performance of the sensor, and there are inaccuracies or distortions in the measurement data, resulting in errors and uncertainties in the environmental perception results. Therefore, the environment sensing technology of underwater ROV based on artificial side line is proposed. According to the artificial side line structure, the underwater terrain environment perception principle is constructed, and each characteristic parameter is counted. The underwater acoustic sensor network communication model was constructed, the sound field disturbance based on the artificial side line normalization was analyzed, and the impact response function and response time approximation were calculated by using the transmitting array and receiving array to obtain the fixed point of the underwater moving target and realize underwater ROV environment perception. The experimental test results show that under the artificial side line, the predicted value of ROV environment sensing technology is always floating around the true value curve, and the positioning performance is good. Under different conditions, the measurement error decreases to less than 1%. When controlling the environment perception position of underwater ROV, the obtained coordinates are basically consistent with the expected coordinates, realizing the perception of underwater terrain environment and target positioning, which has a wide application prospect for underwater exploration, Marine scientific research, diving operations and other fields. In future studies, the statistical methods of characteristic parameters will be continuously optimized to improve the accuracy and stability of statistical results. At the same time, the introduction of additional environmental parameters will also be considered to more fully reflect the changes in the underwater environment.

## 1 Introduction

The underwater environment is characterized by high pressure and low temperature, which will limit the performance of the sensor and the accuracy of data acquisition. At the same time, due to the absorption and scattering characteristics of seawater, underwater acoustic transmission is widely used in underwater environment. However, factors such as the complex flow of seawater, terrain changes and target interference further increase the difficulty of underwater environment perception. ROV is an unmanned submersible operated remotely, which is usually used for exploration and operation in underwater environments such as deep sea and coastal areas. Accurate environmental perception is essential for safe operation, effective exploration and scientific research of underwater ROVs [1–3]. However, due to the complexity and randomness of the underwater environment, such phenomena as dark swell often occur, and the use of side-scan sonar to present the detection results in the form of images [4,5] is often limited, resulting in errors and uncertainties in the environmental perception results. Therefore, in order to improve the environment perception ability of underwater ROV and correct the measurement error, it is of great significance to study a new ROV environment perception technology.

Lateral line system is an important sensing system of aquatic organisms, especially fish, which can make them better feel the dynamics of water and environmental changes. It is a new development trend and research hotspot in the field of underwater robot to develop the artificial side line on the ROV.

Qin H in 2020 proposes the autonomous environment and target perception of underwater vehicles [6]. Autonomous underwater robots rely on underwater cameras and sonar to perceive the surrounding environment, including underwater image processing, target detection and tracking. For underwater optical environment perception, the limited visual range of the camera significantly hinders the reception of detection information.; To improve the underwater image quality and extract more matching feature points. In order to achieve accurate registration between images, underwater stitching technology is also involved, and a fusion algorithm is implemented to eliminate artificial stitching traces. Experimental results show that this framework can not only keep the details of underwater images, but also accurately match more feature points for registration and stitching. For underwater acoustic image environment perception, the underwater robot is usually equipped with mechanical scanning imaging sonar to avoid obstacles and multi-target tracking. Han X et al. in 2022 proposes the wearable sensor-based underwater environmental emotion evaluation model [7]. Underwater sensor network technology and equipment have developed rapidly. Underwater Internet of things devices have been widely used in energy investigation, environmental index detection, and disaster event monitoring. Transferring massive underwater data to the cloud for processing and analysis has become the mainstream processing paradigm, and cloud computing has become the mainstream computing paradigm. The preparation strategy of elastomer coated hydrogel fiber for stable optical sensing proposed in this paper opens up a new method and approach for developing low-cost and high-sensitivity water flow sensors. At the same time, the design of wearable intelligent devices is analyzed to evaluate the emotional perception evaluation scheme of underwater environment. The distributed edge computing is introduced to bear part of the pressure of cloud computing, and an underwater sensor network data acquisition and sensing scheme based on edge prediction is proposed, which realizes the conversion from underwater acoustic communication transmission part of underwater data to data prediction transmission, thus reducing the energy consumption caused by underwater acoustic communication. The model established in this paper effectively reduces the energy consumption of the sensor, ensures the accurate data transmission, and can respond to the potential demand in time, which is obviously superior to the existing schemes. Wang S et al. in 2022 proposes the biomimetic whisker sensor model based on frictional electric nanogenerator for underwater use [8]. The generator’s high sensitivity to stimulation is adopted to obtain the motion state of underwater objects and sense the hydrodynamic trajectory generated by underwater moving objects. Although the triboelectric nanogenerator has high sensitivity, in underwater environment, due to the influence of the water medium and the contact effect between the electrode and the water, the sensitivity of the sensor will be reduced, resulting in the perception of the hydrodynamic trajectory is not accurate enough.

In the process of application, although the above method can reduce the environmental perception error, the correction effect when the results are distorted still needs to be further verified. Therefore, in order to ensure the reliability of environmental perception of underwater topography, this paper proposes an underwater ROV environmental perception technology based on artificial side line. This technology mainly enhances and corrects the images of underwater detectors during the detection and imaging process, so as to ensure the accuracy of the output results of terrain environmental perception. The principle of underwater terrain environment perception is constructed by using the structure of artificial side line, and each characteristic parameter is counted. Data transmission and target location are realized by the communication model of underwater acoustic sensor network. By utilizing artificial sidelines, more accurate and reliable environmental information can be obtained, improving the safety, operability and efficiency of subsea ROVs.

## 2 Environmental perception of underwater ROV with artificial siding

### 2.1 Artificial siding structure

In underwater environments, it is often difficult for ROVs to accurately determine their position due to the lack of obvious landmarks. The construction of artificial sidelines can provide visible and fixed reference objects to help the ROV accurately locate and navigate. The artificial sideline structure can be designed according to the special properties of the underwater environment, with higher adaptability and flexibility, and can better conform to the underwater terrain and target characteristics, thus improving the accuracy and accuracy of data acquisition.

**Fig 1:**
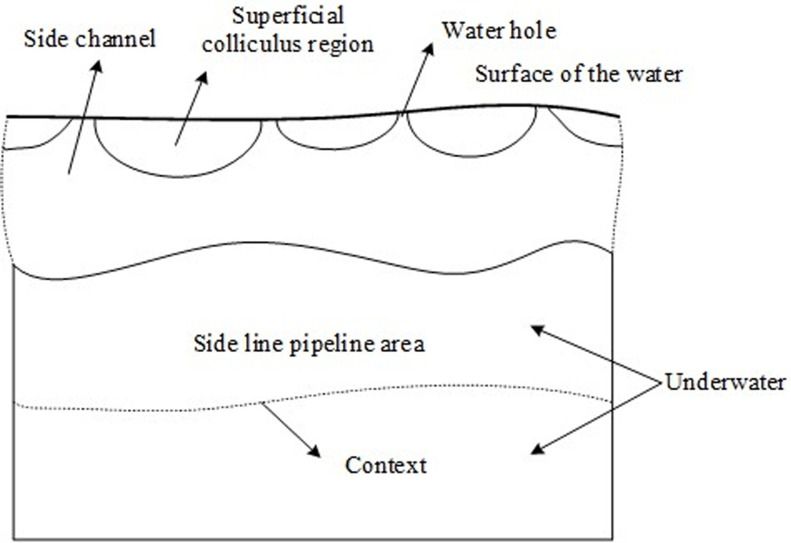
Structure diagram of artificial siding.

Artificial lateral line is a special sensory organ, which is closely related to the behaviors of fish, such as predation, avoiding enemies, group swimming, reproduction, etc. fish rely on the hydrodynamic characteristics of the body surface to obtain their movement information [9]. The artificial side line structure is one of the important components to form the side line of the fish. The development of the artificial side line system using the bionic method can enable the mechanical fish and diving robot to sense the surrounding environment sensitively, timely and accurately [10]. The biological characteristics of the artificial side line structure are deeply discussed, which lays a solid theoretical foundation for the bionics and design of the artificial side line structure. When the ROV conducts underwater exploration or search missions, the presence of artificial lateral lines can provide clear references and marks to help the ROV identify and locate mission targets and improve the accuracy and efficiency of target detection and recognition. The lateral line structure consists of the surface colliculus region and the lateral line pipeline region. They sense the movement of water through sensing neurons respectively. Their distribution, the number and shape of sensing cells and other factors determine their functions. The artificial side line structure is shown in Fig 1.

It can be seen from Fig 1 that the side line pipeline area is located at the side line pipe in the artificial side line structure. It is very sensitive to acceleration, can sense higher frequency components, and can sense pressure changes. It is a sensor similar to a pressure gradient sensor. The surface colliculus region is a conduit composed of a large amount of colloidal liquid, which is closely connected to the surrounding water.

The implementation of artificial siding system provides a way for the analysis of marine hydraulic performance and a new method for the senses of underwater vehicles and other similar fish. In the deep sea, fish can identify obstacles through artificial siding system, so as to avoid obstacles and hunt or avoid them.

### 2.2 Error correction of underwater terrain environment perception

#### 2.2.1 Principle of underwater terrain environment perception.

In the process of underwater terrain environment perception, it is necessary to detect the size, size, position and underwater surface state of underwater reefs and obstacles, so as to provide basis for underwater navigation, underwater condition judgment, landform change, etc. [11].

In the process of underwater terrain environment perception, measure the three-dimensional coordinates $\left(x,y,z\right)$ of the detection points according to the multi beam emitted by the detection instrument, where *x* represents the longitude of the environmental perception measurement point, *y* represents the latitude of the environmental perception measurement point, and *z* represents the depth value of the underwater environmental perception position; Data filtering method is used to process the data obtained from underwater terrain environment perception to form a depth data matrix, complete the fusion of measurement point data, form the overall depth environment perception result describing the underwater area, and form the environment perception image result, that is, to complete the environment perception of underwater terrain.

In the process of underwater terrain environment perception, the particularity of the underwater environment, the existence of undercurrent and other factors lead to the difference in resolution between the output underwater terrain map and the actual underwater map [12], and there is also a certain error in the relative rotation angle, resulting in the distortion and deviation of the underwater terrain image measured by environment perception. Therefore, if the underwater situation is judged directly based on the measured image obtained in this case, the accuracy of the judgment results will be poor. To improve accuracy, the characteristic parameters of underwater terrain environment perception need to be further analyzed.

#### 2.2.2 Characteristic parameter statistics of underwater terrain environment perception.

The mean elevation, roughness, information entropy and difference entropy are the key parameters to reflect the diversity of underwater terrain features. Average elevation describes the horizontal plane of the overall underwater terrain, roughness represents the roughness of the terrain, and information entropy and difference entropy show the variation and irregularity of the terrain elevation. These parameters can be combined to comprehensively describe the morphology, complexity and diversity of underwater terrain [13]. The dispersion degree, smoothness degree and central tendency of underwater terrain elevation are described by the above characteristic parameters, so as to obtain the inherent characteristic attributes of underwater terrain.

Suppose that the longitude and latitude span of an environment sensing area is x×y, and the elevation value at the coordinate $\left(m,n\right)$ is represented by $z\left(m,n\right)$. The detailed definition of the characteristic parameters of the underwater terrain is as follows:

$H_{h}$ refers to the mean value of underwater terrain elevation, and its expression formula is:


$$H_{h}=\frac{\sum_{i=1}^{m}\sum_{j=1}^{n}z\left(m,n\right)}{x\times y}$$ (1)

Assuming that the surface area and projected area of underwater terrain are expressed in $S_{a}$ and $S_{b}$ respectively, and the roughness of underwater terrain is expressed in $C_{cd}$, the expression formula is:


$$C_{cd}=\frac{S_{a}}{S_{b}}$$ (2)

Underwater terrain information entropy is expressed by $X_{xd}$, and its expression formula is:


$$X_{xd}=\sum P_{ij}\times C_{cd}$$ (3)

Where: $P_{ij}$ represents the elevation value of underwater terrain after normalization. Assuming that the underwater terrain difference entropy is expressed in $H_{e}$, the expression formula is:


$$H_{e}=\sum X_{xd}\times Y_{gh}\times D_{FF}$$ (4)

Where: $Y_{gh}$ represents the difference probability, and $D_{FF}$ represents the underwater terrain difference value.

The above characteristic parameters cover all the characteristics that affect the accuracy of environmental perception in the whole process of underwater terrain environment perception. The selection of underwater terrain feature parameters can respond sensitively to environmental changes. When the underwater terrain changes, the statistics of the above parameters can quickly respond to changes in the underwater environment and provide real-time terrain feature information. By monitoring and analyzing these parameters, the ROV can sense changes in the underwater terrain in time, helping to accurately capture environmental changes and the appearance of obstacles, and improving the accuracy of environmental perception.

## 3 Environment aware mobile target location method for underwater ROV

### 3.1 Construction of underwater acoustic sensor network communication model

In the underwater acoustic sensor communication network, the underwater acoustic communication model between each node in the sensor and the underwater moving target cannot be described. In order to fully describe the multi dynamic new underwater acoustic sensor network communication, it is necessary to select the shortest baseline for impact response in each cycle [14–15]. In order to prevent data loss in underwater ROV environment aware underwater acoustic sensor network communication, at the signal receiving end of the underwater acoustic sensor network communication model, an underwater acoustic sensor network communication model is constructed by using equalization technology to prevent interference from other force majeure factors.

The environment aware underwater acoustic sensor network communication model of underwater ROV collects the data of underwater moving targets at the information acquisition end of the underwater acoustic sensor network communication model, processes and analyzes the data according to the decision-making noise reduction coding algorithm [16], and calculates the ability of the underwater acoustic sensor network to target positioning in combination with the artificial side line. Then the information data acquisition can be expressed as:


$$R(k)=y(k)\times J_{k}\times J_{m}\times W_{f}$$ (5)

Where y(k) represents the collected information of the communication model of the underwater acoustic sensor network, $J_{k}$ represents multiple communication channels, $J_{m}$ represents the amount of noise interference in the communication process of the underwater acoustic sensor network, and $W_{f}$ represents the output data of the communication model of the underwater acoustic sensor network.

After the analysis and processing of the collected data, the data of the underwater acoustic sensor network communication model is effectively identified, and the interference code in the data is calculated for the interference elements of multiple communication channels [17–18]. According to the underwater acoustic sensor network communication model, after the data information collection, analysis and processing, the output value is equal to the input value of the underwater acoustic sensor network communication path. According to the above process, the communication model of underwater acoustic sensor network is constructed.

### 3.2 Acoustic field disturbance of underwater moving target based on artificial side line normalization

**Fig 2:**
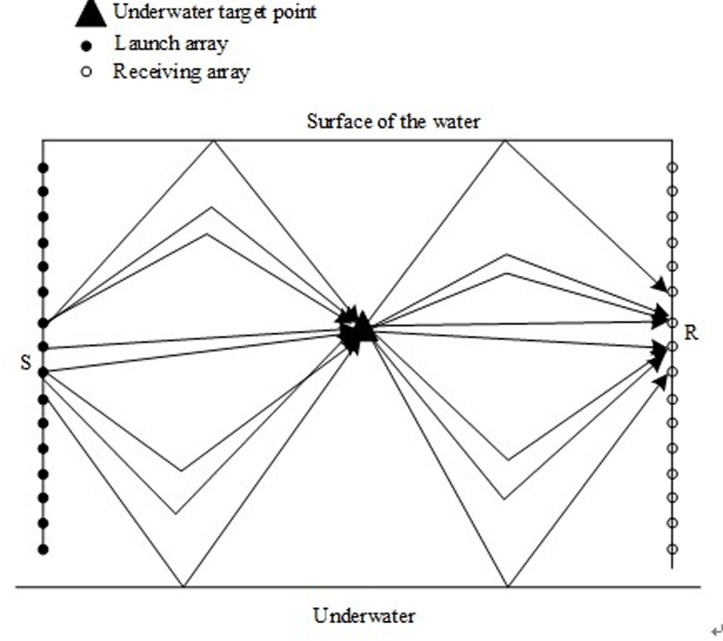
Positioning diagram of underwater moving target.

In the process of normalizing the sound field disturbance of the underwater moving target based on the artificial side line, the transmitting array and receiving array of the artificial side line are used to form the target sound field disturbance [19], and the underwater moving target is positioned, as shown in Fig 2.

The transmitting array based on man-made side line underwater moving target positioning is composed of multiple transmitting array elements, and the receiving array is composed of multiple receiving array elements, which together constitute a vertical array. When no underwater moving target is found, the received signal is:


$$O_{mn}=A_{ss}⊗B_{mn}+C_{nm}⊗g_{k}$$ (6)

Where $B_{mn}$ represents the transmitting signal of the *m* th transmitting array element on the *n* th receiving array element, *k* represents the transmitting time of the underwater moving target array element, $A_{ss}$ represents the transmitting signal of the transmitting array element, $C_{nm}$ represents the corresponding function of the underwater acoustic sensor network communication model,  ⊗  represents the neural network operation, and $g_{k}$ represents the underwater noise interference.

Suppose that the underwater moving target at point $\left(p,q\right)$ has *M* incident sound lines and *N* outgoing sound lines, the impact response function of the incident sound line to the underwater moving target is $X_{Y}$, the response time is $T_{i}$, the impact response function of the outgoing sound line to the underwater moving target is $V_{as}$, and the response time is ${\tilde{T}}_{j}$. Take any group of incoming and outgoing sound lines, and calculate the approximation of the impact response function and response time as follows:


$$D_{f}=\frac{M\times N}{X_{Y}\times T_{i}\times \left(p,q\right)}\times V_{as}\times \alpha$$ (7)

Where, *α* represents the emission angle of the above selected two emission lines. The impact response functions of the incident sound line and the outgoing sound line under the impact response obtained above are superimposed to obtain the approximation function of the two, and the response function of the underwater moving target is obtained [20]. Due to the fact that there are many paths and the energy on many paths can be neglected during the underwater propagation of artificial side lines, assuming that the underwater environment remains unchanged and the influence of noise is not considered, the received signal can be expressed as:


$$P_{pq}=\left(g_{pq}+g_{pq}\prime \right)⊗\left(n(t)+n(t{)}^{\prime }\right)$$ (8)

Where $P_{pq}$ represents the received signal of the underwater moving target. Taking values of the output signal $g_{pq}$ and input signal $g_{pq}\prime$ of the underwater moving target respectively, the pulse signal disturbance of the underwater moving target can be expressed as n(t) and $n(t{)}^{\prime }$. In order to calculate the sound field disturbance of the underwater moving target, the sound field disturbance caused by the above incoming and outgoing sound lines is normalized based on the artificial side line, and the following formula is obtained:


$$B_{ij}=\left(n(t)+n(t{)}^{\prime }\right)/P_{pq}$$ (9)

Where $B_{ij}$ represents the sound field disturbance of the underwater moving target.

### 3.3 Environment awareness technology for underwater ROV

**Fig 3:**
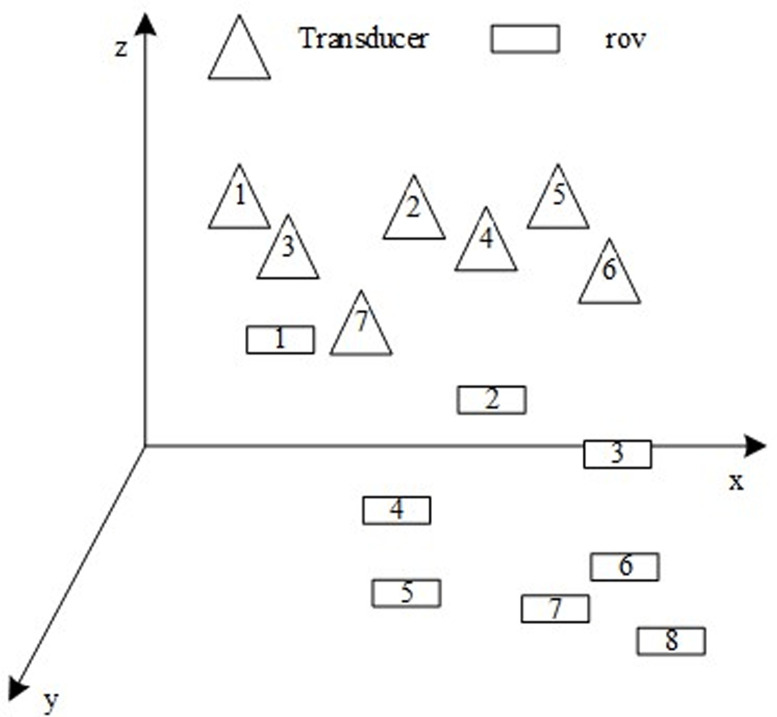
Positioning diagram of underwater moving target.

In order to ensure the stability and accuracy of the environment sensing technology for underwater ROV, according to the normalization processing results of the acoustic field disturbance of underwater moving targets based on artificial side lines, an underwater target location algorithm is designed. The hydrophone is installed on the underwater moving target, and the underwater ultrashort baseline signal is transmitted through the displacer. On the premise of ensuring the stable power supply of the displacer, the signal sent by the underwater moving target is received through the hydrophone to improve the stability of the whole technology operation. The schematic diagram of underwater ROV environment sensing technology is shown in Fig 3.

As shown in Fig 3, the coordinate system is constructed in the current moving area of the underwater moving target, and the position of the underwater moving target can be estimated by the distance between the signal sent by the underwater moving target transmitter and the displacement detector. The position of the transmitter used to transmit the positioning signal is consistent, and the positioning signal is determined by the displacer.

Combined with the least square method, the position of the positioning point of the underwater moving target is obtained as follows:


$$W_{ZX}={\left(B^{T}B\right)}^{-1}\times B_{i}j$$ (10)

Where, *B* represents the target point of underwater moving target positioning, and $B^{T}$ represents the transposition of the target point. Above, the design of underwater moving target location algorithm is completed, and the environment sensing technology of underwater ROV based on artificial siding is realized.

## 4 Experimental test analysis

In order to test the effect and applicability of the environment sensing technology of underwater ROV based on man-made side line in this paper, this technology is applied to the terrain and geomorphic environment sensing of a certain sea area. The equipment used for the measurement is a side-scan sonar, using a RESON Seabat7125 multi-beam sounder with an operating frequency of 200 kHz, a beam Angle of 1.5° x 120°, a number of beams of 256, and a maximum single beam depth of 7500m. The underwater acoustic equipment is suitable for complex underwater environmental conditions, can be applied to underwater sounding, terrain scanning and other tasks, has a wide range of applications, and can meet the needs of underwater environment sensing technology testing and research. The detector is installed at the bottom of the detection ship to obtain the underwater terrain environment perception image and complete the perception test of this technology.

In the underwater environment, due to water flow, underwater equipment movement and other factors will lead to data changes, the use of convolutional neural network parameter sharing and migration invariance characteristics, can make the training of the model more efficient and can cope with the input data of small translation changes, help to improve the robustness of the model. In the environment perception test, the initial learning rate of the convolutional neural network experiment is set to 0.001. the maximum number of iterations is 200, and the convolution kernel size is 5 ×  5. The water depth is 500m, the attenuation coefficient is 0.40, and the density of underwater low mass is 2.5g·cm^−3^. Other parameter information is shown in Table 1.

**Table 1:** Experimental parameter information setting table.

Parameter name	Specific value or content	Parameter name	Specific value or content
Underwater sound velocity	1762m/s	Distance of transceiver array	2km
Weight value among network layers	0.55	Sound source emission center frequency	0.5kHz
Center pulse width of sound source emission	80ms	Small system	Digital processing chip

**Fig 4:**
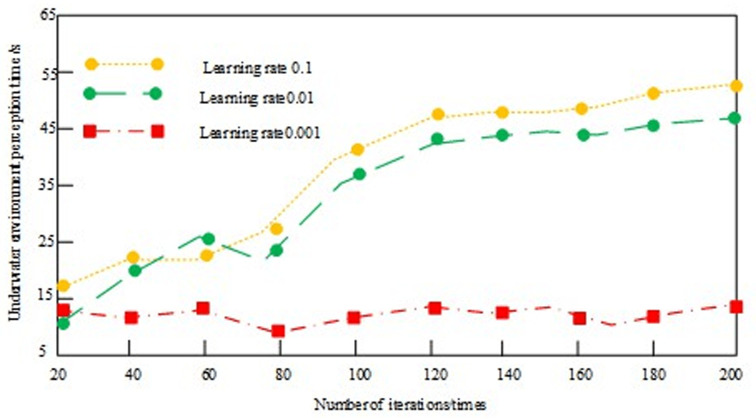
Comparison of environmental perception time results at different learning rates.

In order to verify the suitability of the learning rate parameter setting of the convolutional neural network, the performance of the method was verified within 200 iterations, taking the environment perception time as an indicator. The experimental results are shown in Fig 4.

According to the results in Fig 4, there are obvious differences in the environment perception time under different learning rates, which indicates that the learning rate parameters have an important impact on the training efficiency and convergence speed of the model. When the learning rate is 0.001, the environment perception time is shorter, indicating that the model can converge faster or obtain better performance.

**Fig 5:**
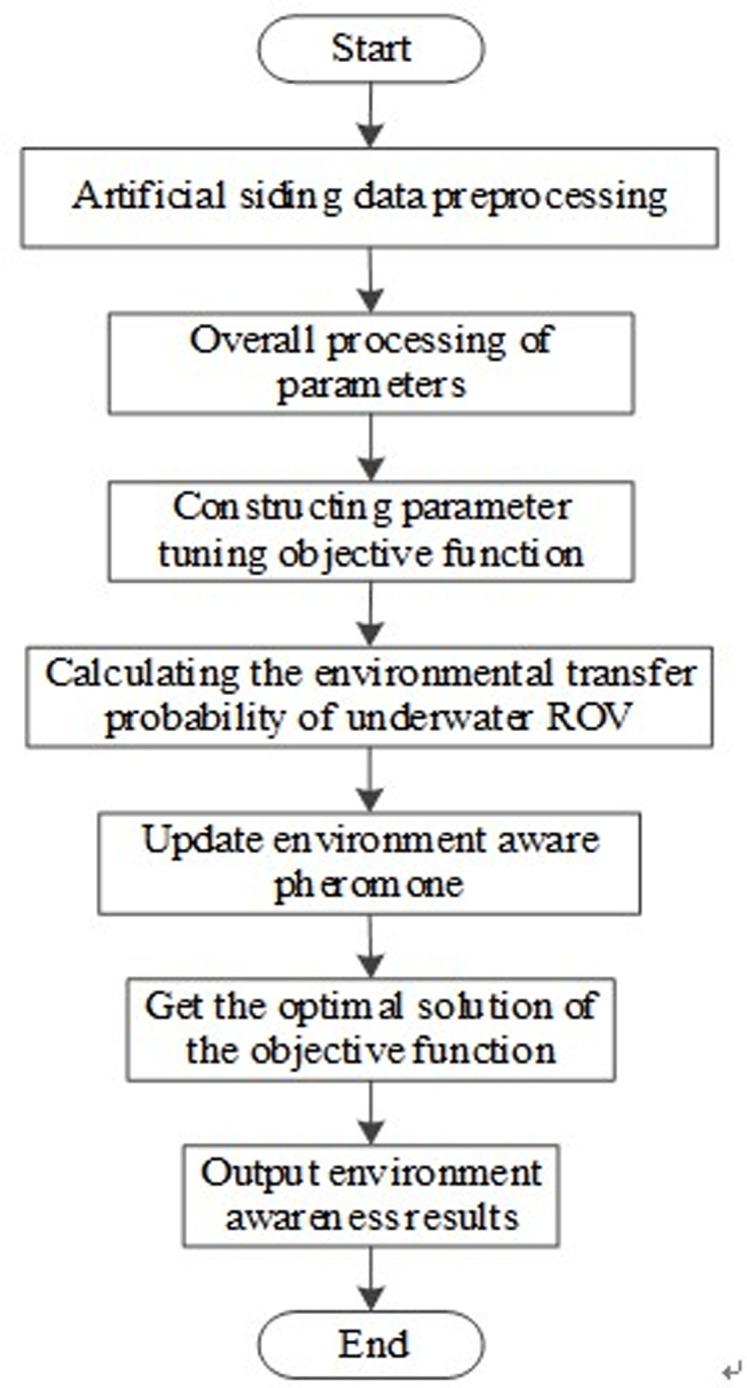
Experimental process of underwater ROV environment awareness technology.

The experimental process of environment perception technology of underwater ROV based on artificial side line is shown in Fig 5.

**Fig 6:**
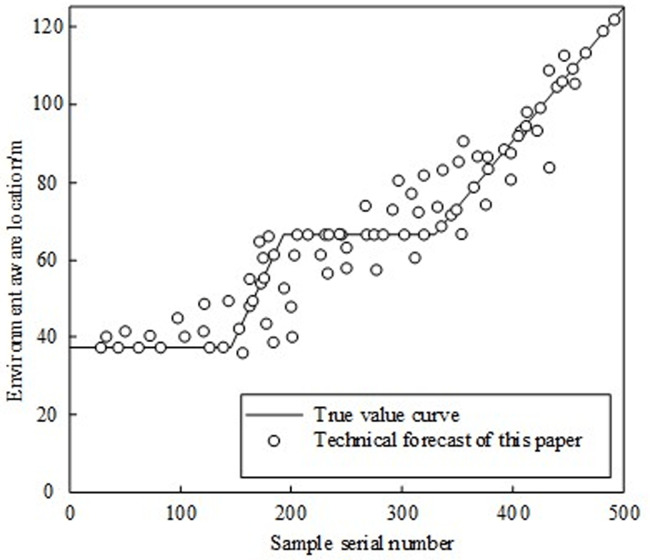
Prediction results of underwater ROV environment perception technology.

Assuming that there is a mismatch of sound velocity profile between the underwater environment and the simulation environment, the real sound velocity profile data measured by the RESON Seabat7125 multi-beam sounder is used to simulate and generate the received signal data by combining the underwater channel transmission model and the sound propagation principle. 1000 groups of received signal data generated by simulation are preprocessed as actual data under the artificial siding. The environment sensing technology of underwater ROV based on artificial side line is used to predict the received signal data, and the results are shown in Fig 6.

It can be seen from Fig 6 that for the received signal data, the predicted value of the underwater ROV’s environment sensing technology always fluctuates around the real value curve, indicating that the underwater ROV’s environment sensing technology has good positioning performance under the artificial side line. 500 groups of received signal data are used to locate the underwater ROV’s environment sensing technology, so as to reduce the impact of the underwater ROV’s environment sensing technology on the underwater mobile target positioning performance.

In order to test the environmental perception performance of the technology in this paper, 25% of the mean and median color deviation angle, the best and worst cases of the measured image are used as the test conditions, and the error correction results of the method in this paper under the four conditions are obtained, as shown in Table 2.

**Table 2:** Calibration performance test results of environment Aware Technology.

Condition category	Error results/%	
	Terrain image error before environment perception technology correction	Terrain image error corrected by environment perception technology
Mean value of color deviation angle	6.50	0.88
	6.53	0.85
Median	6.95	0.95
	6.35	0.89
Optimal	6.15	0.90
	7.25	0.86
Worst	7.29	0.84
	7.38	0.82

According to the test results in Table 2, it is concluded that the obtained terrain image error before correction by environmental perception technology is 6.8% under four conditions; After the correction of environmental perception technology, the measurement error results under the four conditions are reduced to less than 1%. Therefore, the technology in this paper has good performance of environment perception error correction, and can ensure that the underwater terrain environment perception results obtained after correction are closest to the actual results.

**Fig 7:**
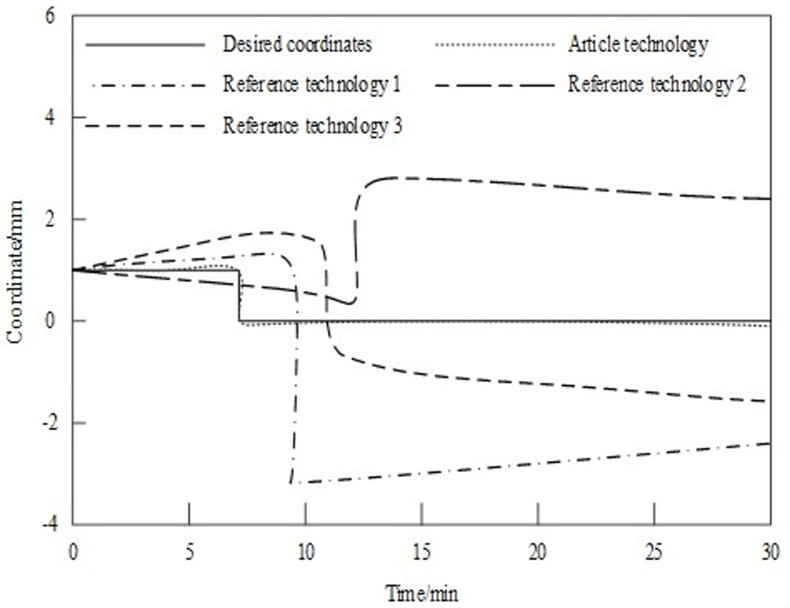
Coordinate position control results.

On this basis, position control tests are carried out using the technology in this paper, the autonomous environment and target perception technology of underwater vehicle in reference [6] (referred to as “reference technology 1”), the underwater environment emotion evaluation technology based on wearable sensor in reference [7] (referred to as “reference technology 2”), and the underwater bionic whisker sensor technology based on triboelectric nanogenerator in reference [8] (referred to as “reference technology 3”), respectively. The comparison results are shown in Fig 7.

As can be seen from Fig 7, When reference technology 1, reference technology 2 and reference technology 3 are used to control the position of the underwater vehicle’s environment perception, there is a gap between the coordinates in the control process and the expected coordinates, but the coordinates obtained by the technique in this paper are basically consistent with the expected coordinates in the position control test. Because the technology of this paper is based on the normalization processing results based on the artificial side line of the acoustic field disturbance of the underwater moving target, The underwater target location algorithm is designed. The hydrophone is installed on the underwater moving target, and the underwater acoustic ultra short baseline signal is transmitted through the displacer. Based on this, the motion control parameters are adjusted, and the accuracy of the position control results is improved.

## 5 Conclusion and prospect

### 5.1 Conclusion

This article introduce artificial lateral-line system structure, research the ROV underwater environment perception. In this study, the artificial side line structure is combined with the underwater terrain environment perception principle to build the corresponding underwater acoustic sensor network communication model, which can better realize the underwater environment perception and positioning, and has high practicality and applicability. The artificial side line is used to normalize the disturbance of underwater moving target, which improves the detection and location accuracy of underwater moving target. The impact response function and response time approximation are calculated by using the coordination between the transmitting array and the receiving array, so as to obtain the fixed point of the underwater moving target. The technology in this paper has a good performance of environmental perception error correction, which can ensure that the underwater terrain environmental perception results obtained after correction are the closest to the actual results, and the underwater terrain image results are almost consistent with the original image results. When the technique is used to test the position control, the obtained coordinates are basically consistent with the expected coordinates.

### 5.2 Discussion and prospect

This study introduced the artificial lateral line structure unique, and combine the principle of underwater terrain environment perception can be more comprehensive and accurate implementation of the underwater environment awareness and positioning, so as to improve the performance of the method as a whole. However, due to the underwater environment, including ocean currents, tides, seabed topography, water quality and other factors, the sound wave propagation under the water is affected by water temperature, pressure, salinity, etc., the sound velocity profile, resulting in different performance of positioning and ranging results in the complex acoustic environment, thus affecting the accuracy of artificial side shield technology. In practical application, due to the complexity and variability of underwater environment, the statistics of characteristic parameters may be interfered by many factors, such as water temperature, salinity, flow rate, etc. These factors lead to deviations in the statistical results of characteristic parameters, thus affecting the accuracy of environmental perception. Therefore, in subsequent studies, statistical methods will be further optimized to improve the accuracy and stability of statistical results. At the same time, the introduction of additional environmental parameters will also be considered to more fully reflect the changes in the underwater environment.
